# Supplementary material for: Trait-Based Method of Quantitative Assessment of Ecological Functional Groups in the Human Intestinal Microbiome
Source: Biology (Basel). 2023 Jan 11;12(1):115. doi: 10.3390/biology12010115 (PMC9855786; doi:10.3390/biology12010115)
Supplement: Supplementary file 1 [file biology-12-00115-s001.zip › Document S1.pdf]

Reasons for choosing functional groups

In the human gut, the anaerobic microbial community decomposes and ferments dietary fiber that has escaped digestion in the upper intestinal tract. The decomposition and fermentation of these substrates require the contribution of various groups of microorganisms linked into the trophic chain, which performs the decomposition of food polysaccharides into smaller fragments that are then fermented into short-chain fatty acids, namely, acetate, propionate and butyrate, as well as gases, mainly H_2 and CO_2 [24].

Despite the fact that almost all members of the microbial community are somewhat involved in the fermentation of carbohydrates, bacteria that specialize in complex polysaccharides can be distinguished. It is these members who provide the first stages of fermentation of complex substrates [24,34]. Our methods do not yet allow us to label them as a distinct functional group; however, we have included them in the general reconstruction scheme in order to obtain more comprehensive understanding of the processes occurring in the ecosystem.

Our main interest was in the production and interconversion of low molecular weight compounds in the ecosystem. We chose H_2 as a key metabolite for our reconstruction, since its accumulation in the system inhibits the reoxidation of NADH, thereby reducing the yield of ATP and short-chain fatty acids [35]. Thus, its concentration indirectly affects the entire community. Since it is inevitably produced at most stages of substrate fermentation, it must be constantly removed from the environment in order to ensure a more complete oxidation of substrates [39].

There are two main ways in our community to remove H_2 from the system. The first of these is acetogenesis [33]. Acetate is produced by reductive acetogenesis from H_2 and CO_2 as an energy source by various types of epithelial cells [20]. Acetate can also be effectively used by some groups of anaerobic bacteria as a growth substrate or for conversion to butyrate, which is a more beneficial short-chain fatty acid for the host. In our community, *Marvinbryantia formatexigens* is an acetogen [23]. Due to acetogenesis, *Marvinbryantia formatexigens* has a unique capacity in our community to use CO_2 for its growth.

The second way to utilize hydrogen is sulfate reduction [30]. In the microbial community under study, this function is performed by *Desulfovibrio piger*, representatives of this species absorb hydrogen and make use of the entire production of sulfate by the rest of the bacteria, converting it into hydrogen sulfide. If the product of acetogenesis, acetate, is mostly useful both for bacteria in the community and for the host organism, then hydrogen sulfide, on the contrary, is rather toxic [27].

A source of sulfate is required for sulfate reduction to proceed. In the human large intestine, it can be mainly obtained from bile acids, sulfur-containing amino acids and mucin breakdown products [36]. Thus, sulfate reduction may depend on the activity of mucin-decomposing bacteria. All these factors, namely, the dependence on the decomposition of mucin, the toxicity of hydrogen sulfide and the role in the utilization of hydrogen, determined our choice to distinguish these bacteria into a separate functional group of sulfate reducers.

Moreover, the stability of the mucin layer is of critical importance for maintaining the health of the host, therefore, considering mucin-decomposing bacteria as a distinct

functional group has not only ecological sense, but also potential biomedical importance [10,26,32].

In addition, in order to assess the potential effect on the health of the host, we chose butyrate-producers as a functional group. Butyrate can serve as a food source for host colonocytes, and also has an anticarcinogenic effect [25,28,37].

General principles for selecting TDGFs

Next, we will describe the general principles that allowed us to select TDGFs in this way.

If we need to estimate the abundance of organisms that yield certain product, we must find such a reaction that is strictly necessary for the work of only this metabolic pathway, its “bottleneck”.

In the case we need to evaluate the degradation of a certain substrate, it is quite rare that only one enzyme is responsible for its degradation. Therefore, the selection of TDGFs for functional groups, which are responsible for degradation process, is difficult.

However, it is worth noting the situation when one has to deal with complex substrates that are decomposed through a cascade of reactions. In this case, it is necessary to look for such a reaction, without which the ultimate degradation of the substrate is impossible. Examples of the described principles implementation are demonstrated in the section "Key enzymes that determine the ecological function of microorganism groups".

Selection of TDGFs for functional groups

The key metabolic pathway for acetogens is the Wood - Ljungdahl pathway, which allows them to use hydrogen as an electron donor and carbon dioxide as an electron acceptor for acetate biosynthesis. Figure S1 D shows this path divided into two branches: "Eastern" and "Western".

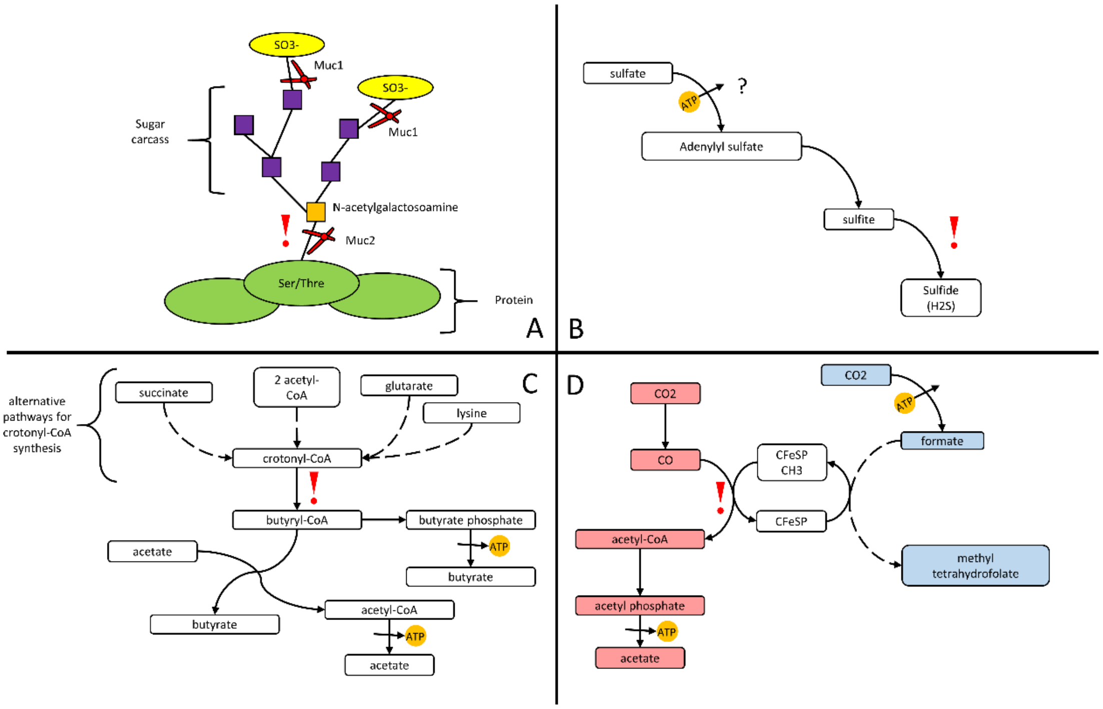

Figure S1. The reactions that are key for expressing the traits of respective functional groups. A - scheme of mucin decomposition, B - sulfate reduction, C - butyrate synthesis, D - acetogenesis. Red rectangles depict the west branch and blue rectangles depict the east branch of the Wood - Ljungdahl pathway. The exclamation point marks the reaction, which is catalyzed by an enzyme that can serve as a TDGF of the respective functional group. The scissors indicate the reaction of destruction of the covalent bond.

The enzymes in the eastern branch of the pathway that are involved in the conversion of CO_2 to methyl tetrahydrofolate are common in most organisms, although they are expressed at much higher levels in acetogenic organisms. Enzymes of the western branch of the pathway are specific for this functional group and their loci are located close to each other in the genomic sequence [31]. Therefore, we chose four enzymes of the western branch of the pathway as TDGFs: carbon monoxide dehydrogenase, acetyl-CoA synthetase, and both subunits of the corrinoid iron-sulfur protein, which belong to the western branch of the Wood - Ljungdahl pathway. It is worth noting that all the selected TDGFs in our case are located next to each other on the genomic sequence, therefore they are interchangeable (in our study, we mainly used carbon monoxide dehydrogenase).

Dissimilatory sulfate reductase, which is present in *Desulfovibrio piger* in the studied community, was chosen as a TDGF of sulfate reduction. As shown in Figure S1 B, it is the terminal enzyme of the sulfate dissimilation pathway [35].

The synthesis of butyrate can be performed in at least four currently known pathways (Figure S1 C) [38]. The main metabolic pathway of synthesis is its formation from acetyl-CoA, while the rest of the pathways are mostly minor. However, they all eventually intersect at the stage of crotonyl-CoA synthesis. Butyryl-CoA dehydrogenase (or crotonase) converts it to butyryl-CoA. Then the pathway of butyrate formation divides into kinase (butyrate-phosphate formation) and transferase (exchange of coenzyme A with acetate). We chose butyryl-CoA dehydrogenase as a TDGF of butyrate synthesis, since it unites all the aforementioned pathways. In addition, we believe that enzymes that carry out the reaction of the kinase pathway can nonspecifically participate in similar reactions of coenzyme A metabolism, which is also the factor that determined our choice.

Mucins are complex glycoproteins [21]. All of them are highly O-glycosylated and therefore the significant part of the mucin peptide is protected from proteolytic degradation due to the presence of these glycan chains [29]. Therefore, we decided to focus specifically on the enzymes associated with the cleavage of O-glycans as TDGFs for mucin-decomposers.

Mucin O-glycans are covalently bonded to the main chain of the polypeptide through N-acetylgalactosaminyl Ser / Thr residues. O-glycosylation of mucin glycoproteins occurs in regions rich in the amino acid residues Pro / Thr / Ser (mucin domain) and the resulting common molecular structures of mucin glycoproteins are believed to be in the form of bottlebrushes. O-glycans are synthesized by sequential addition of monosaccharides through the organized action of glycosyltransferases in mucin-producing cells, the number of possible modifications is very large and the structure of mucin O-glycans is highly heterogeneous. Despite the fact that they are usually divided into several groups depending on "main" types (mainly core 1, core 2, core 3 and core 4 are present in nature), the set of enzymes for their degradation will vary significantly for different mucins [21]. Thereby, we decided to choose N-

acetylgalactoseaminidase as one of the TDGFs, which destroy the bond between the first monomer of the sugar backbone and the amino acid residue. This connection is present in any mucin, which means that by choosing a TDGF this way, we will be able to obtain the broadest possible information about mucin degradation (Figure S1 A).

We also have noticed the curious defense system of mucins from intestinal bacteria. It is known that secreted colon mucins have oligosaccharide side chains that are more sulfated than side chains in areas of the gastrointestinal tract with fewer bacteria. Accumulated data indicate that sulfation of mucins makes them less vulnerable to degradation by bacterial glycosidases [22]. However, some bacteria possess mucin-desulfating sulfatases, which desulfate sulfomucin, allowing glycosidases to access and act on mucins. Therefore, we also chose this enzyme as a TDGF (Figure S1 A).

All selected single TDGFs used in the work are shown in Table S3.

Table S3. Single TDGFs of functional groups

Functional group	Single TDGF	Enzyme	EC-number	Identification number NCBI
Acetogens	acet1	carbon monoxide dehydrogenase	1.2.7.4	WP_040782480.1
	acet2	acetyl-CoA synthetase	2.3.1.169	SDF10942.1
	acet3	corrinoid iron-sulfur protein (large subunit)	2.3.1.169	SDF11010.1
	acet4	corrinoid iron-sulfur protein (small subunit)	2.3.1.169	SDF10976.1
Sulfate-reducers	sulfat1	dissimilation type sulfite reductase	1.8.99.5	WP_040369683.1
Butyrate-producers	but1	butyryl-CoA dehydrogenase	1.3.8.1	ERI80036.1
Mucin-decomposing bacteria	muc1	sulfatase	3.1.6.-	WP_005775657.1
	muc2	alpha-N-acetylgalactoseaminidase	3.2.1.49	BBP48024.1

Verification of correct selection of TDGFs for functional groups

Next, we made the choice of TDGFs that allow us to assess the abundance of functional groups for high-throughput sequencing data of intestinal microbiota communities.

Since we exactly know from the literature the affiliation of the representatives of the synthetic microbial community to functional groups, we decided to make sure that the TDGFs were chosen correctly. To do this, we aligned each TDGF against the available reference genomes of the studied microorganisms (available in Supplementary Material 5 at data\references). The alignment results are shown in Table S4.

Table S4. Results of the alignment of TDGF sequences on the reference genomes of microbiota representatives. The color indicates the functional groups: but - butyrate-producers, muc - mucin-decomposing bacteria, acet - acetogens, sulf - sulfate-reducers. Score values greater than 200 for the alignments of TDGFs to the reference genomes are colored according to the functional groups.

Functional group	Organism	Score values for aligning TDGFs				
		**but1**	**muc1**	**muc2**	**sulfat1**	**acet1**
	Bacteroides ovatus	141	**663**	**273**	31	28
	Bacteroides uniformis	147	**640**	**279**	34	27
muc	*Bacteroides thetaiotaomicron*	142	**669**	**281**	31	28
	Bacteroides caccae	144	**677**	**286**	31	27
	Barnesiella intestinihominis	150	**569**	**327**	29	29
	Akkermansia muciniphila	28	**243**	**868**	33	28
but	*Roseburia intestinalis*	**614**	29	25	58	33
	Eubacterium rectale	**574**	**248**	48	50	36
	Clostridium symbiosum	**737**	**239**	57	60	34
	Faecalibacterium prausnitzii	**463**	197	32	30	26
acet	*Marvinbryantia formatexigens*	28	**238**	47	28	**1275**
sulf	*Desulfovibrio piger*	25	27	31	**795**	34
	Escherichia coli	152	27	39	28	28
	Collinsella aerofaciens	26	30	45	30	27

In general, the TDGFs showed their specificity, but the TDGFs of mucin-decomposing bacteria are much more widespread than the representatives of their functional group. This is primarily because mucin degradation is a complex multi-stage process, and various organisms can participate in it at certain stages, and only few specialists are able to carry it out completely. Since we have selected a limited number of key reactions as TDGFs, it is not surprising that they are also found in other representatives of the microbiota, especially in bacteroides, that are specialists in the decomposition of polysaccharides. Due to the greater specificity of Alpha-N-acetylgalactoseaminidase in comparison with sulfatase, we decided to use it as a TDGF of the functional group of mucin-decomposing bacteria further.
