# Supplementary material for: Trait-Based Method of Quantitative Assessment of Ecological Functional Groups in the Human Intestinal Microbiome
Source: Biology (Basel). 2023 Jan 11;12(1):115. doi: 10.3390/biology12010115 (PMC9855786; doi:10.3390/biology12010115)
Supplement: Supplementary file 1 [file biology-12-00115-s001.zip › Document S2.zip!/Document S2/Manual.docx]

This archive is a set of scripts and instructions, which allow you to fully reproduce the method of quantitative assessment of ecological functional groups. It is assumed that you have all the necessary programs installed in your working environment, such as fastq-dump, Trinity, and tblastn. We used Python version 3.8.3.

The bioinformatic analysis pipeline carried out in the article is presented in Figure 1.

**Figure 1.** The pipeline of bioinformatic analysis of the abundance of ecological functional groups. Stage 1 involves the metatransriptome assembly, which is carried out using the Trinity platform; the alignment on stage 2 is carried out in tblastn; the contig abundance is calculated in stage 3 using Kallisto, and finally stages 4 and 5 are carried out by the scripts we have developed using Python programming language.

***Stage 0 - Download and preprocess the data***

To get started, you need to create a project directory.

mkdir Project

cd Project

Copy all files from the archive to this folder.

The “intermediate files” folder contains all the intermediate files that can be created during the execution of the pipeline. You can use them to test how it works.

The data will be downloaded to the “data” folder under the directory below.

cd data

The analyzed metatranscriptomics data were taken from [1] and have the following BioProjectID identifiers: SRP092478, SRP092476, SRP092461, SRP092458, and SRP092453. All of these datasets are presented in table 1.

| Project | Archive | index | Diet |
| --- | --- | --- | --- |
| SRP092478 | [SRR4841988](https://trace.ncbi.nlm.nih.gov/Traces/sra?run=SRR4841988) | FR1 | fiber-free/fiber-rich |
|  | [SRR4841989](https://trace.ncbi.nlm.nih.gov/Traces/sra?run=SRR4841989) | FR2 |  |
|  | [SRR4841990](https://trace.ncbi.nlm.nih.gov/Traces/sra?run=SRR4841990) | FR3 |  |
| SRP092476 | [SRR4841891](https://trace.ncbi.nlm.nih.gov/Traces/sra?run=SRR4841891) | FP1 | prebiotic/fiber-free |
|  | [SRR4841892](https://trace.ncbi.nlm.nih.gov/Traces/sra?run=SRR4841892) | FP2 |  |
|  | [SRR4841893](https://trace.ncbi.nlm.nih.gov/Traces/sra?run=SRR4841893) | FP3 |  |
| SRP092461 | [SRR4841487](https://trace.ncbi.nlm.nih.gov/Traces/sra?run=SRR4841487) | P1 | prebiotic |
|  | [SRR4841488](https://trace.ncbi.nlm.nih.gov/Traces/sra?run=SRR4841488) | P2 |  |
|  | [SRR4841489](https://trace.ncbi.nlm.nih.gov/Traces/sra?run=SRR4841489) | P3 |  |
| SRP092458 | [SRR4841407](https://trace.ncbi.nlm.nih.gov/Traces/sra?run=SRR4841407) | F1 | fiber-free |
|  | [SRR4841408](https://trace.ncbi.nlm.nih.gov/Traces/sra?run=SRR4841408) | F2 |  |
|  | [SRR4841409](https://trace.ncbi.nlm.nih.gov/Traces/sra?run=SRR4841409) | F3 |  |
| SRP092453 | [SRR4838357](https://trace.ncbi.nlm.nih.gov/Traces/sra?run=SRR4838357) | R1 | fiber-rich |
|  | [SRR4838358](https://trace.ncbi.nlm.nih.gov/Traces/sra?run=SRR4838358) | R2 |  |
|  | [SRR4838359](https://trace.ncbi.nlm.nih.gov/Traces/sra?run=SRR4838359) | R3 |  |

Download all data to the current directory.

bash downloadSRR.sh

gunzip -k *.gz

In order for Trinity to work with the downloaded files, we need to use fastq-dump (splitSRR.sh), then rename the resulting files and put them into separate directories.

bash splitSRR.sh

bash renameSRR.sh

The sequences of the trait-determining genetic features (TDGFs) have already been pre-downloaded and are located in the “TDGFs” directory.

For further work, it is necessary to remove contamination and low-quality reads.

Downloading human/mouse genomes:

bash downloadGenome.sh

Contamination control:

bash bowtie2-build.sh

bash bowtie2.sh

Quality control:

bash fastp.sh

***Stage 1 - Assembling the transcriptome***

*De novo* transcriptome assembly is performed using Trinity for working with RNA-Seq data obtained from a non-model organism. Since “samples.txt” contains only three columns, Trinity uses the mode for unpaired reads.

*De novo* transcriptome assembly takes a long time and always leads to a slightly different result, thence the “trinity_out_dir” directory contains exactly the version of the assembly that we used while preparing the article.

Trinity --seqType fq --samples_file samples.txt --max_memory \ 10G --CPU 6

cd ../

***Stage 2 - Search for TDGFs in the transcriptome assembly***

Next, it is necessary to align all amino acid sequences of enzymes used as TDGFs against the transcriptome assembly. The list of used TDGFs is contained in the file “tdgfs_name.txt”, which serves as an input file for “tblastn.sh”. To reproduce the results of the article, the following TDGFs should be used: acet1, sulfat1, but1, muc2 (they are used by default in “tdgfs_name.txt”).

bash tblastn.sh

Using the “findContigID-def.py” script, you can select contig IDs that contain the target TDGFs into the “ContigID_markername.txt” file. By default, only those contigs that pass the Score threshold for alignment equal to 250 are selected for this file.

python findContigfID-def.py

***Stage 3 - Obtaining data on abundance of transcripts***

We assume that the nucleotide sequences of the contigs in the assembly correspond to the sequences of the transcripts. Therefore, to obtain data on the abundance of transcripts, the Kallisto program is used. The sequences are indexed for the transcriptome assembly. Then pseudo-alignments (an optimized algorithm based on the use of k-mers) are carried out for each sample per indexed assembly. The default parameters are used throughout. Data on the abundance of transcripts are presented in the form of TPM (transcripts per million) [2].

mkdir abundance

kallisto index -i abundance/Trinity.index \ data/trinity_out_dir/Trinity.fasta

bash kallisto_quant.sh

***Stage 4 - Search for TPM values for TDGFs contigs***

The following script allows you to select TPM values for the lists of contig IDs (obtained during Stage 2) containing TDGFs:

bash find.sh

***Stage 5 - Processing the results***

Next, you need to format the output files in such a way that all contigs are numbered as textN_M, where “text” is the name of the functional group, “N” is the TDGF number from “data/TDGFs/<your_tdgf_type>” (only one TDGF must be used for each functional group), and “M” is the contig number.

python TPMformater-def.py

The output file contains a table that can be easily interpreted by the user and is used as the input file for StatUp-def.

python StatUp-def.py

Using the “StatUp-def.py” script, the TPM values of individual TDGF-containing contigs are summarized for each biological sample (F1, F2, R1…). Next, the TPM values are combined into samples by types of diets (3 samples in each diet type). For such samples, mean TPM values are calculated.

The output file is a figure depicting the abundances of a particular functional group calculated by two methods. You can change the abundance calculation formula (as shown in our article) and the analyzed functional group by changing the options inside the script.

***References***

1.	Desai, M. S.; Seekatz, A. M.; Koropatkin, N. M.; Stappenbeck, T. S.; Martens, E. C. Article A Dietary Fiber-Deprived Gut Microbiota Degrades the Colonic Mucus Barrier and Enhances Pathogen Article A Dietary Fiber-Deprived Gut Microbiota Degrades the Colonic Mucus Barrier and Enhances Pathogen Susceptibility. **2016**, 1339–1353. https://doi.org/10.1016/j.cell.2016.10.043.

2.	Wagner, G. P.; Kin, K.; Lynch, V. J. Measurement of MRNA Abundance Using RNA-Seq Data: RPKM Measure Is Inconsistent among Samples. *Theory Biosci.* **2012**, *131* (4), 281–285. https://doi.org/10.1007/s12064-012-0162-3.
